# Hypomethylation-induced regulatory programs in T cells unveiled by transcriptomic analyses

**DOI:** 10.3389/fimmu.2023.1235661

**Published:** 2023-09-27

**Authors:** Memnon Lysandrou, Panagiota Stamou, Dionysia Kefala, Chryso Pierides, Maria Kyriakou, Nikolaos Savvopoulos, Panayiota Christofi, Anastasia Papadopoulou, Evangelia Yannaki, Paul Costeas, Alexandros Spyridonidis

**Affiliations:** ^1^Bone Marrow Transplantation Unit and Institute of Cell Therapy, University of Patras, Rio, Greece; ^2^The Center for the Study of Haematological and other Malignancies and Karaiskakio Foundation, Nicosia, Cyprus; ^3^Gene and Cell Therapy Center, Hematopoietic Cell Transplantation Unit, Hematology Department, “George Papanikolaou” Hospital, Thessaloniki, Greece

**Keywords:** regulatory T-cells, hypomethylating agents, HLA-G, RNA-Seq, ScRNA-seq, IDO-1

## Abstract

Regulatory T cells (Tregs) are essential mediators of tolerance mitigating aberrant immune responses. While naturally occurring Treg (nTreg) development and function are directed by epigenetic events, induced Treg (iTreg) identity and mechanisms of action remain elusive. Mirroring the epigenetic circuits of nTregs, we and others have used hypomethylation agents (HAs) to *ex vivo* convert T cells into iTregs (HA-iTregs) and further showed that the suppressive properties of the HA-iTregs are predominantly confined in an emergent population, which *de novo* expresses the immunomodulatory molecule HLA-G, consequently providing a surface marker for isolation of the suppressive HA-iTreg compartment (G^+^ cells). We isolated the HA-induced G^+^ cells and their G^−^ counterparts and employed high-throughput RNA-sequencing (RNA-seq) analyses to uncover the G^+^-specific transcriptomic changes guiding T cells toward a regulatory trajectory upon their exposure to HA. We found a distinct transcriptional upregulation of G^+^ cells accompanied by enrichment of immune-response–related pathways. Although single-cell RNA-seq profiling revealed regulatory G^+^ cells to have molecular features akin to nTregs, when assessed in conjunction with the comparative transcriptomic analysis and profiling of secreted cytokines against the non-suppressive G^−^ cells, FOXP3 and other T-helper signatures appear to play a minor role in their suppressive phenotype. We found an ectopic expression of IDO-1 and CCL17/22 in G^+^ cells, denoting that *in vitro* exposure of T cells to HA may well unlock myeloid suppressor genes. This report provides transcriptional data shaping the molecular identity of a highly purified and potent HA-iTreg population and hints toward ectopic myeloid-specific molecular mechanisms mediating HA-iTreg function.

## Introduction

Regulatory T cells (Tregs) are essential mediators of immunological tolerance mitigating deviant or overt immune responses as illustrated by *in vitro* and *in vivo* studies and early phase clinical trials (1). Tregs can be encountered as naturally occurring cells *in vivo* (nTregs) or may be *in vitro* induced upon modulation of conventional T cells (iTregs) (2–5). While nTreg development and function are meticulously directed and orchestrated by epigenetic events ensuring specific and stable demethylation status of key genes, including the Forkhead Box P3 (FOXP3) transcriptional factor, iTregs identity and mechanisms of action remain largely ambiguous and probably highly dependent on the inherent variability of their generation protocol (3, 4, 6, 7). Seeking to mimic the epigenetic circuits of nTregs, several groups including ours have used hypomethylation agents (HAs) to *ex vivo* convert murine or human T cells into iTregs (HA-iTregs) (5, 8, 9). In an attempt to identify the exact molecular mechanism guiding the function of such murine HA-iTregs, Choi et al. elegantly demonstrated that their *in vitro* and *in vivo* suppressive abilities occur independently of FOXP3 (9). Human leukocyte antigen G (HLA-G) molecule is a well-known immunomodulatory molecule that shields the “semi-allogeneic” fetus from maternal immune rejection during pregnancy and is epigenetically repressed succeeding prenatal life (10). Naturally occurring HLA-G^+^ T cells are encountered in low percentages in human peripheral blood as part of the physiological repertoire of suppressor, distinct from nTregs, T cells and can putatively expand when needed to preserve immune tolerance (11–13). In our previous work, we hypothesized that HLA-G has an operative role in the suppressive action of HA-iTregs (5, 11). Indeed, by using the HA decitabine (Dec), we robustly and consistently produced human HA-iTregs with *in vitro* and *in vivo* suppressive activity that have now entered a phase I–II clinical trial against Graft-versus-Host disease (EudraCT 2021-006367-26), and showed that their suppressive function is mediated to a large extent but not exclusively by the HLA-G molecule (5, 14, 15). HA-induced *de novo* expression of HLA-G on T cells remains functionally stable upon removal of the HA as well as in pro-inflammatory environment (5). More importantly, we showed that the suppressive properties of the HA-iTregs are strictly confined in the HLA-G–expressing cellular compartment, henceforth, providing a surface marker for isolation of the suppressive HA-iTregs (termed G^+^ cells).

Aiming to unveil the immunosuppressive mechanisms of HA-iTregs and elucidate the epigenetic networks guiding T cells toward a regulatory trajectory upon their exposure to HA, we employed high-throughput analyses to uncover the G^+^-specific transcriptomic changes. Interestingly, we link *de novo* expression of established myeloid regulatory genes, such as indoleamine 2,3-dioxygenase 1 (IDO-1) with HA-iTregs emergence.

## Materials and methods

### Generation of HA-induced iTregs

Peripheral blood mononuclear cells (PBMCs) were obtained from healthy volunteers under informed consent and approval from the local ethics committee (Protocol Number: 5832). T cells were isolated by negative magnetic cell selection (MACS Pan-T Cell Isolation Kit II Human MiltenyiBiotec, Germany) except for the flow cytometry-based validation experiments where CD4^+^-selected T cells were used as starting material (RosetteSep Human CD4+ T-cell enrichment cocktail, StemCell Technologies, Canada), then activated for 3 days in the presence of anti-CD3/CD28 beads (Dynabeads Human T-Activator CD3/CD28, Gibco) at a 1:1 bead:cell ratio and subsequently incubated in the presence of 50U/mL human recombinant IL-2 (rhIL-2 PeproTech or Miltenyi Biotec) with 5 μM or 7.5 μM Dec (Sigma-Aldrich, Germany) for three additional days. Control T cells were treated with phosphate-buffered saline (PBS) solution and further denoted as PBS-treated controls.

### Flow cytometry and cell sorting

Flow cytometry was performed on BDFACS Canto II Flow Cytometer (BD Biosciences, USA) and data analysis was performed on FlowJo v10. Cell sorting was performed using BDFACSAria III (BD Biosciences) by gating on single viable HLA-G^+^CD4^+^ and HLA-G^−^CD4^+^ cells reaching a purity of sorted cell populations over 95%. The following monoclonal anti-human antibodies were used: HLA-G-PE (clone MEM- G9, Sigma-Aldrich), CD4 FITC/PerCP/APC-Cy7 (clone RPA-T4, BD Biosciences or BioLegend) and CD3-FITC (clone UCHT-1, BD Biosciences), IDO-1-Alexa Fluor 647 (clone V50-1886, BD Biosciences), CD45RA-PE-Cy7 (clone HI100, BioLegend), CD62L-V450 (clone DREG-56, BD Biosciences), CD25-PE (clone M-A251, BioLegend), CD127-PerCP-Cy5.5 (clone HIL-7R-M21, BD Biosciences), and GITR-BV421 (clone V27-580, BD Biosciences). Dead cell exclusion was performed with Zombie Aqua/NIR Fixable viability dye (BioLegend) or Far-Red Live/Dead Fixable dead cell stain kit (Invitrogen, USA). Fixation was performed using BD Phosflow Fix Buffer (BD Biosciences), and permeabilization using perm buffer (PBS, Triton 0.5%, and BSA 0.5%). Fluorescence minus one (FMO) control was used to set the threshold of positivity for HLA-G, CD45RA, CD62L, CD25, CD127, and GITR.

### RNA sequencing and single-cell RNA sequencing

Total RNA was isolated from five paired samples (G^+^/G^−^) using the miRNeasy Micro kit (Qiagen, Germany), according to manufacturer’s instructions and was used for bulk RNA-seq. Illumina-compatible libraries were prepared according to manufacturer’s instructions and next generation sequencing (NGS) (paired-end and strand-specific) was performed on an Illumina HiSeq2000. For scRNA-seq, approximately 16.000 sorted G^+^ cells were loaded in a channel of a chromium controller (10× Genomics) for generation of gel-bead-in-emulsions (Greek Research Infrastructure for Personalized Medicine, pMedGR). The sequencing library was prepared using Single Cell 3′ Reagent Kits v3.1 (10× Genomics) and sequenced on an Illumina NextSeq2000.

Bioinformatic analysis for both RNA-seq and scRNA-seq data is described in detail in the **Supplementary Data**.

### Real-time polymerase chain reaction

RNA was isolated from paired sorted G^+^ and G^−^ cells using the RNeasy Micro kit (Qiagen, Germany), cDNA was synthesized (PrimeScript RT Reagent Kit, Takara) and TaqMan assays were performed for HLA-G, IDO-1, CCL22, CCL17, and ABL1. The reaction was performed on a BioRadCFX96, and each cDNA sample was assayed in duplicate. Quantification was performed using the ΔΔCT method and relative expression was calculated based on housekeeping gene (ABL1) expression.

### Multiplex cytokine/chemokine immunoassay and tryptophan/kynurenine quantification

Paired sorted G^+^, G^−^, and PBS-treated controls were plated at 4 × 10^5^/ml in 48-well plates in the presence of IL-2 (100 U/ml) and were either left unstimulated or stimulated with anti-CD3/CD28 beads at a 1:1 bead:cell ratio for 48h. Cell-free supernatants were harvested and stored at −80°C. For cytokine/chemokine quantification, analysis was performed using the Bio-Plex Pro Human Cytokine Th1/Th2 Assay (Bio-Rad, USA) and a custom multiplex magnetic bead-based immunoassay (Protatonce, Ltd), according to manufacturer’s instructions on a Bio-Plex 200 system (Bio-Rad). Tryptophan (Trp) and kynurenine (Kyn) concentrations were assessed with the kynurenine/tryptophan ratio ELISA pack (ImmuSmol), according to manufacturer’s instructions, using the Infinite F50 system (Tecan, Switzerland). All samples were run in duplicates.

### Statistical analyses

Statistical analyses were performed using paired two-tailed standard or ratio *t*-tests using Prism software (GraphPad Software Inc.). *P* values of less than 0.05 were considered significant.

## Results

### G^+^ cells represent a transcriptionally distinct population enriched in immune response pathways

**Figure 1:**
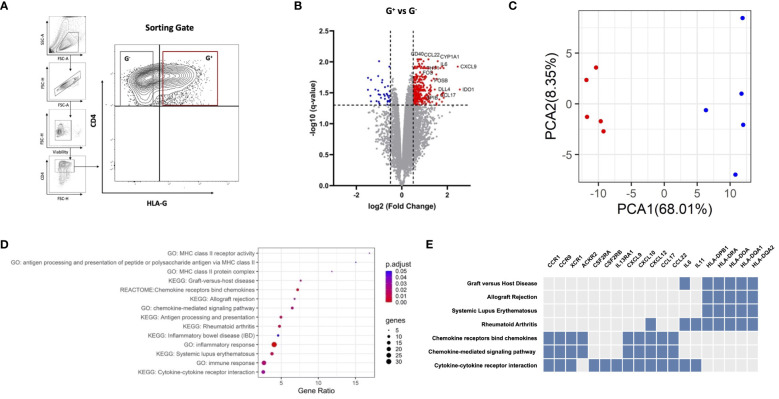
G^+^ cells represent a transcriptionally distinct population enriched in immune response pathways. **(A)** Representative plots of fluorescence-activated cell sorting (FACS) strategy for G^+^ and G^−^ T-cell isolation. Cells were gated on alive singlets with CD4^+^HLA-G^+^ (red gate) or CD4^+^HLA-G^+^ (gray gate) phenotype, where the threshold of HLA-G positivity was set with a fluorescence minus one (FMO) control. **(B)** RNA sequencing of G^+^ T cells against their G^−^ counterparts (*n* = 5 paired samples). Volcano plot of differentially expressed genes (DEGs) in G^+^ T cells, including IDO-1, CCL17, and CCL22. Vertical dotted lines represent |log_2_(fold change)= 0.5, and horizontal dotted lines represent *q*-value = 0.05. Red dots indicate upregulated DEGs, blue dots indicate downregulated DEGs, while gray dots represent genes that do not meet the criteria for differential expression [log_2_(fold change) > 0.5 and *q* < 0.05]. **(C)** Principal components analysis (PCA) plotted using the two main principal components (PCA1-2), depicting the transcriptional profile of the G^+^ population (red) entirely distinct from the G^−^ counterpart (blue). **(D)** Dotplot of the pathway enrichment analysis showing pathways highly enriched in DEGs plotted based on gene ratio (number of DEGs/total genes in the corresponding pathway) using an adjusted *p*-value (p.adjust) threshold of 0.05. **(E)** Clustergram of pathway analysis depicting DEGs associated with enriched pathways of interest. Blue boxes indicate DEGs present in the corresponding pathway whereas gray boxes indicate the absence of the DEG.

We produced HA-iTregs by exposing human peripheral T cells to Dec *in vitro* (*n* = 5) and subsequently sorted the suppressive CD4^+^HLAG^+^ (G^+^) cells and their CD4^+^HLA-G^−^ (G^−^) counterparts to perform comparative transcriptome analysis (**Figure 1A**). A total of 394 genes were identified to be differentially expressed in G^+^ cells as compared to G^−^ cells [DEGs, |log_2_(fold change)| > 0.5 and *q* < 0.05]. Most of the DEGs (*n* = 358) in G^+^ cells were found to be upregulated, which may be either an intrinsic characteristic of these isolated cells or may indicate a more pronounced hypomethylation-induced gene activation in this population (**Figure 1B**). The principal components analysis (PCA) depicted that G^+^ cells are characterized by a distinct gene expression profile that is clearly separate from their G^−^ counterparts (**Figure 1C**). We next conducted *in silico* analysis to identify pathways involved in the transcriptionally distinct HA-induced G^+^ population. Among the highest enrichments in the Kyoto Encyclopedia of Genes and Genomes (KEGG) pathways, the Gene Ontology (GO) molecular function annotations and the REACTOME data repository, several pathways involved in immune responses were uncovered (**Figure 1D**). In particular, chemokine/cytokine receptor interactions and chemokine signaling pathways appear to play a pivotal role in the transcriptional identity of G^+^ suppressive cells. We additionally found numerous pathways related to or containing HLA genes to be enriched in the G^+^ cells (**Figure 1E**). This is not unexpected since HLA-G expression was used to purify the G^+^ compartment. Surprisingly, the HLA-G gene was not identified as an upregulated DEG in the G^+^ cells RNA-seq analysis [HLA-G log_2_(fold change) = 0.3, *p* = 0.019, *q* = 0.087, **Supplementary Figure S1A**], which runs somewhat conflicting to our experimental design. This finding may be related to the shared homology of HLA-G with other DEGs of the HLA family resulting in a high overlap between HLA-G and the other HLA genes or may be due to the variability in HLA-G transcripts between experiments, which may have undermined the statistical power to detect a significant effect on the DEG analysis (**Supplementary Figures 1A, B**). On the contrary, RT-PCR confirmed that HLA-G is truly overexpressed on G^+^ cells (*n* = 5, median fold change-MdFC vs. G^−^ cells 34.66, *p* = 0.0002) (**Supplementary Figure S1C**). Collectively, we show that HA-induced G^+^ cell emergence is closely accompanied by a unique and cohesive transcriptional upregulation of genes involved in immune response pathways.

### The G^+^-specific transcriptome resembles nTreg signatures with an expendable role for FOXP3

**Figure 2:**
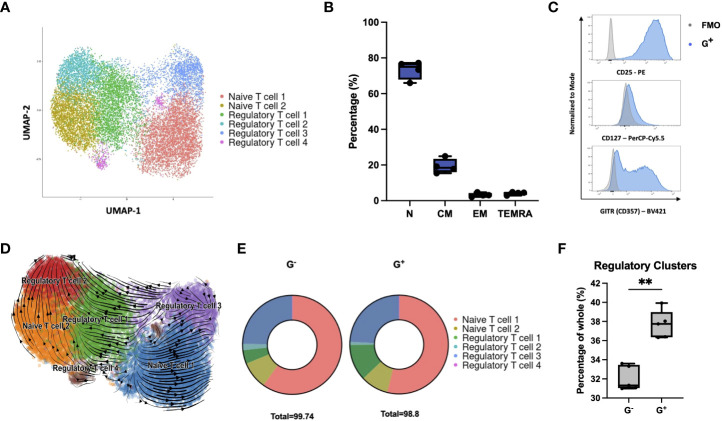
Single-cell RNA sequencing reveals G^+^ cells to be enriched in regulatory signatures. **(A)** Single-cell RNA-seq of G^+^ T cells (n = 15556 cells). Uniform manifold approximation and projection (UMAP) plot showing clustering into regulatory (Regulatory T cells 1–4) and naïve subpopulations (Naïve T cells 1–2) following cell annotation using the scCATCH algorithm. **(B)** Immunophenotypic validation of the early differentiated character of G^+^ cells (n = 4) [N: naïve (CD62L+CD45RA+) median 75%, range 66%–77.2%, CM: central memory (CD62L+CD45RA−) median 18.45%, range 15.4%–24.9%, EM: effector memory (CD62L−CD45RA−) median 3.04%, range 2.1%–4.72%, and TEMRA: terminal effector memory T cells expressing CD45RA (CD62L−CD45RA+) median 4.43%, range 2.87%–4.82%]. **(C)** Immunophenotypic validation of the nTreg characteristics arising from the scRNA-seq data in G+ cells. Representative histograms are shown from one out of four independent experiments. FMO controls (gray) are overlaid to G+ cells (blue) as negative controls (n = 4). **(D)** In silico RNA velocity analysis, projected on UMAP plots, revealing that Regulatory T cell clusters lie downstream of Naïve T-cell clusters in the differentiation trajectory of G^+^ cells. Arrows point toward the predicted course of cell differentiation dynamics while arrow sizes indicate the strength of calculated directionality. **(E)** Deconvolution analysis depicting deconvoluted bulk RNA-seq data of G^+^ and G^-^ T cells using the scRNA-seq–derived dataset. **(F)** Enrichment of G^+^ T cells in Regulatory T-cell cluster signatures compared to G^-^ cells upon deconvolution of bulk RNA-seq data (n = 5, Mann–Whitney test, p = 0.0079). **p ≤ 0.01 and ns, not significant.

We then examined whether G^+^ cells follow a specific trajectory toward categorical T-helper (Th) effector (Th1, Th2, and Th17) or FOXP3^+^ nTreg subsets. Compared to G^−^ cells, transcriptome analysis and cytokine secretion profiling at baseline and stimulated conditions did not reveal a polarization of G^+^ cells toward any trajectory (**Supplementary Figures 2A, B**). Intriguingly, FOXP3 was not found to be differentially expressed in the suppressive G^+^ cells[log_2_(fold change) = 0.26, *p* = 0.0067 and *q* = 0.056] suggesting a dispensable role in the G^+^ phenotype. Since our targeted comparison between the G^+^ and G^−^ compartments does not allow the removal of confounding bias introduced by the HA effects on T-cell fate, we performed single-cell RNA sequencing (scRNA-seq) analysis on isolated G^+^ cells to decipher the underlying heterogeneity of the transcriptional programs of G^+^ cells in an unsupervised manner. Clustering analysis revealed six clusters (**Figure 2A**). Two clusters were clearly demarcated from the rest as a result of the differential expression of a plethora of cell cycle–related genes resulting in a high G2/M gene signature score (**Supplementary Figure S2C**). Cluster annotation using potential marker genes with known cell marker genes from a reference database (CellMatch) allocated four of the clusters to be strongly associated with nTreg cell signatures including FOXP3, IL2RA, and Glucocorticoid-Induced TNFR-related protein-GITR/TNFRSF18 (Regulatory T cell 1–4), while the other two clusters were marked as naïve T cells (Naïve T cell 1–2) (**Figure 2A**, **Supplementary Figure S2C**, and **Supplementary Table S1**). To validate this *in silico* scRNA-seq analysis, we performed flow cytometric analyses (*n* = 4) and showed that G^+^ cells are predominantly early differentiated naïve or central memory cells (**Figure 2B**) and bear a CD25^high^CD127^low/-^GITR^+^ immunophenotype, akin to that of thymic nTregs currently used in clinical practice (**Figure 2C**) (16). By performing *in silico* RNA velocity analysis, we could also uncover a connection between the naïve and regulatory clusters revealing that Regulatory T cell 2 and 3 clusters lie directly downstream of Naïve T cell 2 and 1 clusters, respectively, in the differentiation trajectory of G^+^ cells (**Figure 2D**). Finally, we interrogated our bulk RNA-seq data using the scRNA-seq–derived signature by applying the SCDC deconvolution algorithm and we found a differential composition of regulatory and naïve signatures between G^+^ and G^−^ populations, with the G^+^ fraction to be enriched for regulatory clusters (**Figures 2E, F**). Taken together, although scRNA-seq profiling revealed that regulatory G^+^ cells have molecular features akin to nTregs, when this is assessed in conjunction with the comparative transcriptomic analysis and cytokine profiling against the non-suppressive G^−^ cells, these nTregs signatures seem to play a minor role in the G^+^ identity, thus hinting toward alternate molecular mechanisms instigating the G^+^ suppressive phenotype.

### G^+^-specific transcriptome uncovers ectopic expression of myeloid-specific suppressive genes

**Figure 3:**
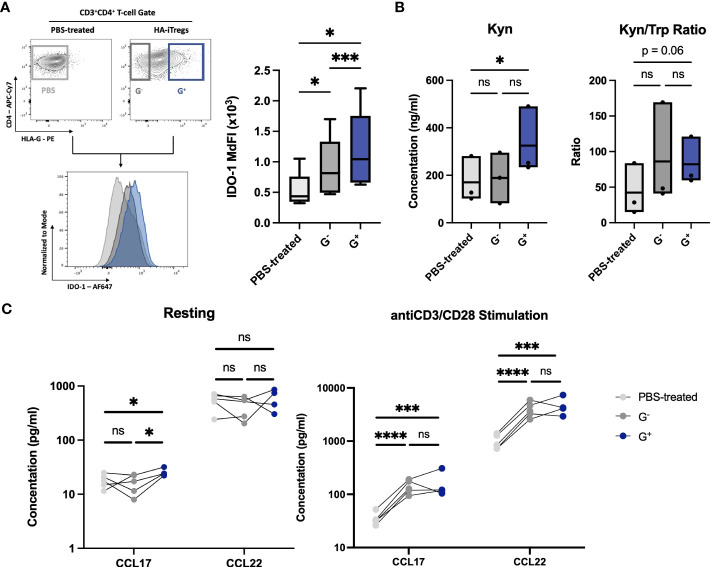
Validation of ectopically expressed myeloid-specific suppressive genes uncovered by G^+^-specific transcriptome. **(Α)** IDO-1 protein expression in G^+^ cells. Representative flow cytometry plots for intracellular IDO-1 detection (left) and collective data of IDO-1 Median Fluorescence Intensity (MdFI) in G^+^ T-cells (blue) over G^-^ T-cells (dark grey) (n=8, p=0.001) and PBS-treated controls (light grey) (n=5, p=0.014) (right). **(B)** Enzyme-Linked Immunosorbent Assay (ELISA) for quantitative analysis of the Tryptophan (Trp) catabolite Kynurenine (Kyn) concentrations (left) and Kyn to Trp ratio (Kyn/Trp) (right) depicting the enzymatic activity of IDO-1 by the means of increased Kyn and Kyn/Trp ratio in G^+^ cell culture supernatants. **(C)** Luminex assay for quantitative assessment of chemokines in cell culture supernatants of G^+^, G^-^ and PBS-treated control cells (n=4-5) at rest (left) or after anti-CD3/CD28 stimulation (right) revealing the increased secretion of CCL17 and CCL22. *p < 0.05, **p ≤ 0.01, ***p ≤ 0.001, ****p ≤ 0.0001, and ns: not significant.

Strikingly, the RNA-seq comparison of G^+^ versus G^−^ cells showed that the top upregulated DEG in G^+^ cells were IDO-1 [log_2_(fold change) = 2.55, *q* = 0.028, **Figure 1B**], a finding which was further validated through RT-PCR (*n* = 4, MdFC = 10.82) (**Supplementary Figure S1C**). IDO-1 was also equally detected in all clusters from the scRNA-seq dataset. IDO-1 is a potent, epigenetically controlled, immunosuppressive molecule mostly associated with myeloid cells and scarcely reported on lymphoid cells (17, 18). This prompted us to further investigate this rather ectopic IDO-1 gene upregulation in T cells by validating its protein expression over both G^−^ cells and paired cells not exposed to Dec (PBS-treated controls). Interestingly, we found a significantly high expression of IDO-1 in G^+^ cells over both G^−^ cells (*n* = 8, *p* = 0.001) and PBS-treated controls (*n* = 5, *p* = 0.0145) (**Figure 3A**). IDO-1 mediates suppression by catabolizing the essential amino acid Tryptophan (Trp) to the immunomodulatory Kynurenine (Kyn) leading to T-cell anergy due to tryptophan starvation and to nTreg induction by Kyn (19, 20). To find a possible functional significance of transcriptional and protein IDO-1 expression in G^+^ cells we measured these metabolites in culture supernatants and calculated the Kyn/Trp ratio as a surrogate marker of IDO-1 enzymatic activity. Indeed, Kyn was found to be highly and significantly produced in G^+^ cells as compared to PBS-treated counterparts (*n* = 3, *p* = 0.03), resulting in a trend toward an increased Kyn/Trp ratio in the G^+^ cell cultures (*p* = 0.06) (**Figure 3B**). Intriguingly, beyond IDO-1 we observed additional mediators of myeloid-driven immune regulation, namely, C–C motif chemokine ligand (CCL) 17 and CCL22, to be among the top 15 upregulated DEGs on G^+^ cells over G^−^ cells [log_2_(fold change) > 1.5]. The transcriptional overexpression of CCL17 and CCL22 chemokines in G^+^ cells was validated by RT-PCR (*n* = 4, MdFC = 4.82, *p* = 0.014 and MdFC = 9.52, *p* = 0.003, respectively) (**Supplementary Figure S1C**). Moreover, the scRNA-seq analysis revealed CCL17 transcripts to be present within all the cluster, with Regulatory T cell 4 cluster displaying the higher level of expression. When compared to G^−^ cells and PBS-treated controls, we found an increased baseline secretion of CCL17 (*n* = 4, *p* < 0.05) from G^+^ cells and excessive amounts of CCL17 (*n* = 4, *p* < 0.001) and of CCL22 (*n* = 4, *p* < 0.001) upon stimulation in both G^−^ and G^+^ supernatants over PBS-treated cells (**Figure 3C**). Taken together, we report that *in vitro* pharmacological hypomethylation of T cells unleashes epigenetically repressed myeloid-specific suppressor genes, such as IDO-1 and CCL17/22.

## Discussion

In this study, we employed G^+^ cells as a model to unravel the molecular underpinnings of the epigenetic reprogramming driving suppressive function in HA-iTregs. Through transcriptome analysis of the suppressive G^+^ cell against the non-suppressive G^−^ cells we show that even though pharmacological hypomethylation takes place in a stochastic fashion, the G^+^ compartment is characterized by a unique gene expression profile that is noticeably distinct from their negative counterparts. These findings are in line with previous work highlighting that isolated suppressive populations of murine and human nTregs and *in vitro*-induced regulatory type 1 (Tr1)- iTregs display a distinct gene expression profile that is clearly separated from their biological counterparts and/or parental cells (3, 4, 6, 7).

Moreover, we identified several pathways implicated in immune responses to be enriched, with chemokines/cytokine receptor interactions and chemokine signaling pathways to be highly represented, which is in agreement with transcriptomic analyses of both isolated suppressive nTregs and iTregs populations (3, 21). We also highlight inherent restrictions of *in silico* tools for accurate HLA inference derived from NGS data that may have hampered previous studies in identifying HLA-G as a key mediator and subsequently marker for iTreg function and identity, respectively (3, 4). Such tools focused solely on HLA genes routinely assessed for clinical HLA typing (HLA-A, -B, -C, -DPA1, -DPB1, -DQA1, -DQB1, and -DRB1-5), thus excluding nonclassical HLA genes, which share homology with genes in the HLA family such as HLA-G, and more specific algorithms to tackle this issue are warranted (22–24).

By performing bulk and single-cell transcriptomic analysis along with cytokine profiling, we show that regulatory G^+^ cells do not follow a specific trajectory toward polarization while displaying selected molecular and phenotypic features of nTregs. These features, nonetheless, appear to play a secondary role in the G^+^ identity, since by comparing the G^+^ suppressive compartment against their equally HA-treated G^−^ counterparts, we show that FOXP3 is not upregulated in G^+^ cells. However, this result does not rule out that FOXP3 is upregulated upon HA treatment as shown by others, but points toward the dispensable role of FOXP3 in the suppressive function of Tregs (8, 9). Similarly, Choi et al. reported that HA-iTregs derived from FOXP3 knockout mice maintained their suppressive abilities *in vitro* and *in vivo* (9), and Lam et al. demonstrated that ablation of FOXP3 in mature nTregs was associated with concurrent demethylated DNA patterns, albeit retention of their suppressive function (21).

Our data hint toward previously unrecognized molecular mechanisms prompting the suppressive HA-iTregs phenotype in G^+^ cells. We report the transcriptional and protein expression of enzymatically active IDO-1 and CCL17/22 by G^+^ cells, which are both atypically expressed in T cells. IDO-1 is a potent regulatory mediator mostly associated with myeloid and mesenchymal stromal cells with implications in feto-maternal tolerance, tumor immune escape, autoimmunity and alloimmune responses such as graft-versus-host disease (18, 25–28). Interestingly, we could identify limited literature on IDO-1 transcriptional expression in T-cells, found to be induced either by IFNa2b or CTLA-4-Fc on CD4^+^ cells (29, 30). Furthermore, a CCL17/22-rich milieu orchestrated by myeloid cells leads to local nTreg chemoattraction, which has been shown to enable tumor immune evasion, prevent autoimmune diabetes and promote donor-specific tolerance (31–34). Collectively, we show that *in vitro* exposure of T-cells to HA may unlock myeloid-specific suppressor genes, but their level compared to naturally expressing myeloid populations remains to be answered. Since these molecules were found predominantly in the suppressive G^+^ compartment, we postulate that they contribute to the suppressive properties of HA-iTregs, something which must be confirmed in future functional studies.

In summary, our study provides compelling evidence that the G^+^ suppressive compartment of HA-iTregs possesses a unique transcriptional profile with prominent regulatory signatures. Our results add to the hitherto literature detaching iTreg function from FOXP3 expression and hint toward novel molecular mechanisms mediating HA-iTregs function, namely the ectopically expressed myeloid-specific molecules IDO-1 and CCL17/22. In conclusion, this report extends our understanding of the molecular features that shape the transcriptional identity of HA-iTregs and provides valuable transcriptional data of a highly purified and potent iTreg population that can be compared with other Tregs (35).

## Data availability statement

The datasets presented in this study can be found in online repositories. The names of the repository/repositories and accession number(s) can be found below: https://www.ncbi.nlm.nih.gov/geo/, GSE229057. https://www.ncbi.nlm.nih.gov/geo/, GSE229170.

## Ethics statement

The studies involving humans were approved by University of Patras Ethics Committee and University General Hospital of Patras Ethics Committee. The studies were conducted in accordance with the local legislation and institutional requirements. The participants provided their written informed consent to participate in this study.

## Author contributions

ML performed the study and wrote the manuscript which was designed and supervised by AS; PS performed sorting experiments for bulk RNA sequencing. CP, DK, MK, PCh, and NS performed additional experiments; PCo supervised sequencing experiments; AP and EY provided critical reviews and edits. All authors contributed to the article and approved the submitted version.
