# Supplementary material for: Hypomethylation-induced regulatory programs in T cells unveiled by transcriptomic analyses
Source: Front Immunol. 2023 Sep 27;14:1235661. doi: 10.3389/fimmu.2023.1235661 (PMC10565652; doi:10.3389/fimmu.2023.1235661)
Supplement: Supplementary file 1 [file DataSheet_1.docx]

Supplementary Material

Hypomethylation-induced regulatory programs in T-cells unveiled by transcriptomic analyses

Memnon Lysandrou^1^, Panagiota Stamou^1^, Dionysia Kefala^1^, Chryso Pierides^2^, Maria Kyriakou^2^, Nikolaos Savvopoulos^1^, Panayiota Christofi^1^, Anastasia Papadopoulou^3^, Evangelia Yannaki^3^, Paul Costeas^2*^, Alexandros Spyridonidis^1*^

^1^Bone Marrow Transplantation Unit & Institute of Cell Therapy, University of Patras, Rio, Greece

^2^The Center for the Study of Haematological and other Malignancies & Karaiskakio Foundation, Nicosia, Cyprus

^3^Gene and Cell Therapy Center, Hematopoietic Cell Transplantation Unit, Hematology Department, “George Papanikolaou” Hospital, Thessaloniki, Greece

*Equal senior authorship

*** Correspondence:** Prof. Alexandros Spyridonidis MD PhD, Bone Marrow Transplantation Unit & Institute of Cell Therapy, University of Patras, Rio, Greece; email: [spyridonidis@upatras.gr](mailto:spyridonidis@upatras.gr)

**Supplementary Data**

**Materials and Methods**

RNA sequencing (RNA-seq):

*Library preparation:*

Total RNA was isolated from 5 paired samples (G^+^ 1-5, G^-^ 1-5) using the miRNeasy Micro kit (Qiagen), according to manufacturer’s instructions and was used for RNA sequencing. Illumina compatible libraries were prepared according to manufacturer’s instructions and Next Generation Sequencing (NGS) (paired-end and strand-specific) was performed on an Illumina HiSeq 2000.

*Data processing & statistical analysis:*

RNA-sequencing data processing was conducted using the InSyBio ncRNAseq tool.^1^ FASTQ files were used, and quality control was checked with FASTQC.^2^ Then trimming of each input fastq file and removal of adapters were performed using Trimmomatic-0.38.^3^ Reads were aligned against the whole human genome (Homo sapiens: hg19 GRCh38) using the HISAT2 tool.^4^ Read counts were calculated using featureCounts tool.^5^ RPKM values were calculated using DESEQ2 tool.^6^ Data were then logarithmized and differential expression analysis was performed using DESEQ2 while correction of p-values for multiple testing was performed using the Benjamini-Hochberg FDR adjustment method. Genes were considered differentially expressed when absolute log2(Fold Change)>0.5 and adjusted p-value (q value or padjust.) was less than 0.05. Principal component analysis (PCA) for feature extraction was performed using the scikit-learn python library version 0.19.2.^7^ Scree test was used to retain an adequate number of principal components to maintain at least the 90% variability of the data set.^8^ Stacked reads plots for HLA isoforms were constructed using IGV version 2.9.2.^9^

*Pathway enrichment analysis:*

Enrichment analysis was conducted using David tool.^10^ This analysis included pathway terms from Reactome data repository,^11^ KEGG^12^ and molecular function annotation from Gene Ontology^13^ and significantly enriched terms were inferred with Benjamini-Hochberg adjusted p-value threshold of 0.05.

Single cell RNA sequencing (scRNA-seq) of sorted G+ cells

*Single-cell RNA capture and sequencing:*

Approximately 16.000 G^+^ cells (1 donor) were loaded in a channel of a Chromium controller (10× Genomics) for generation of gel-bead-in-emulsions. Sequencing library was prepared using Single Cell 3′ Reagent Kits v3.1 (10× Genomics) and sequenced on an Illumina NextSeq2000.

*Data processing:*

FASTQ files were processed using “cellranger count” pipeline from Cell Ranger version 6.1 (10× Genomics) with GRCh38-3.0.0 release as a reference.

*Data quality control:*

Cells outside the thresholds of 200–2500 expressed genes and up to 10% mitochondrial content were removed. Further filtering was performed before unsupervised clustering of cells, in which cells that expressed CD8a were globally removed from downstream analysis (no such cells were found). Only genes expressed in 3 or more cells were considered.

*Data transformation:*

scRNA-Seq data were normalized using the “LogNormalize” method, which normalizes the feature expression measurements for each cell by the total expression, multiplies this by a scale factor (10.000) and log-transforms the result. Final residuals were then scaled to have mean feature expression of 0 and variation of 1 across cells.

*Feature selection, dimensionality reduction, unsupervised clustering and gene signature scoring:*

PCA was conducted to perform feature extraction and dimensionality reduction using the Scree test to keep the principal components which explain 90% of the datasets’ variability. The cells were clustered using the Louvain algorithm^14^ with a resolution parameter of 0.5 having firstly constructed the KNN graph (with k=20, default value) based on the Euclidean distance in PCA space and using Jaccard similarity. Using the clustered data, non-linear dimensional reduction was also performed with Uniform Manifold Approximation and Projection (UMAP).^15^

The scCATCH package was used, which is a single cell Cluster-based annotation Toolkit for Cellular Heterogeneity to identify cluster marker genes and create cluster annotations in a tissue-specific cell taxonomy reference database (CellMatch).^16^ The tissue types: Blood, Peripheral Blood, Plasma, Serum, Umbilical cord blood, Venous Blood were used. For identifying the potential marker genes, the FindAllMarkers function of the Seurat R package was used which finds markers for each of the identity classes. We used logfc.threshold=0.25 and min.pct threshold=0.1. After applying these functions, the clusters were labelled depending on their cell type.

For the Cell Cycle scoring, we used the CellCycleScoring function of the Seurat R package (version 4.3.0.1) using default values for all parameters.

*Trajectory inference:*

The RNA Velocity analysis was performed with the *scVelo* R package (version 0.2.5) using the stochastic model with default values for all parameters. With the stochastic model, we capture the steady states of splicing kinetics for each gene.^17^

*Cell deconvolution analysis:*

The SCDC method and tool was used to deconvolve the initial HLA-G-related RNA-seq using the 6 clusters of cells revealed from the scRNA-seq dataset.^18^ Percentages of cell types in G^-^ and G^+^ cells were compared using the non-parametric Mann-Whitney Test.

# Supplementary Figures


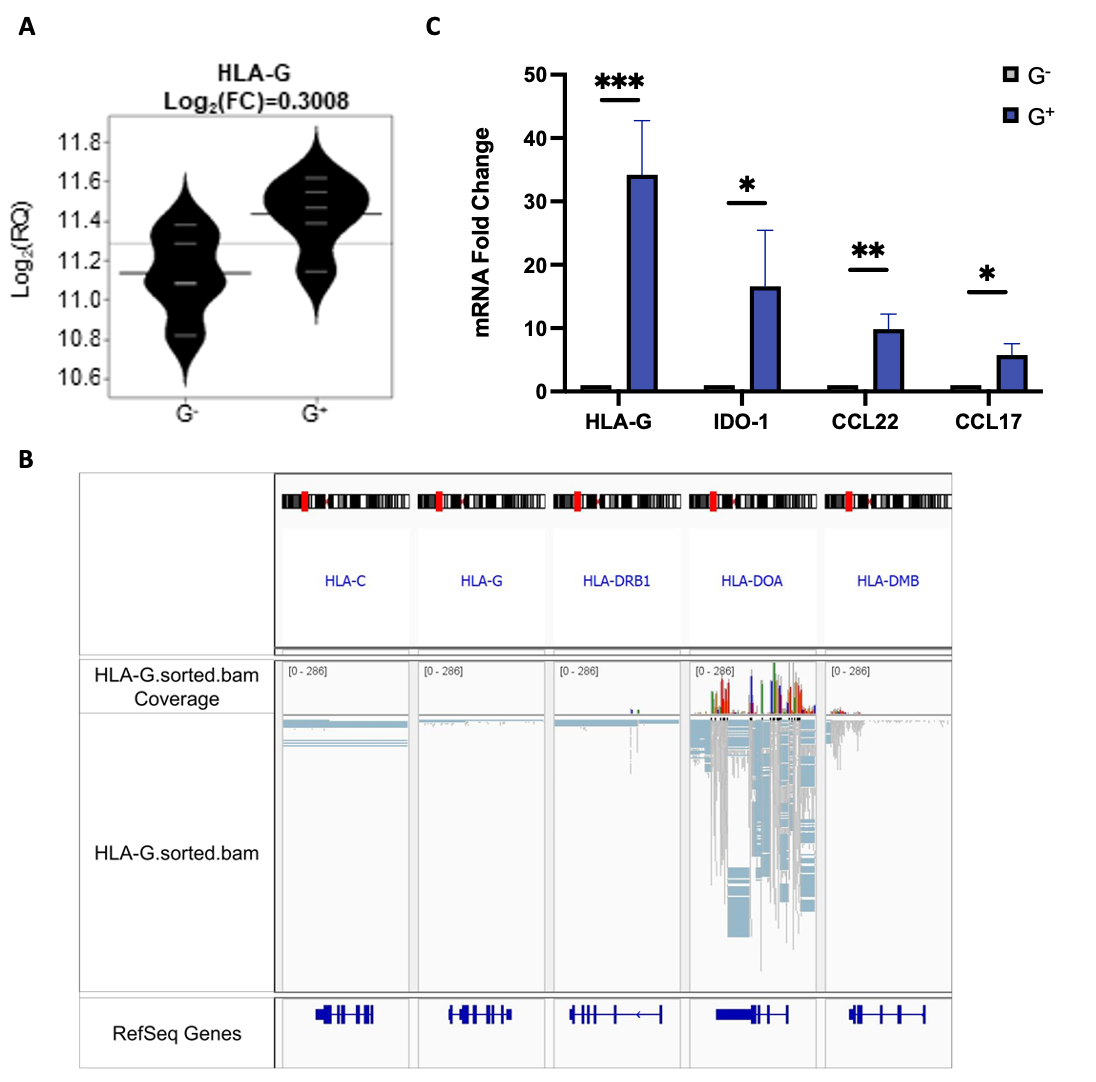


**Supplementary Figure 1.** **Validation of transcriptional upregulation of selected genes and in silico analysis of HLA genes homology.** A) Violin plot depicting HLA-G transcripts of G^+^ and G^-^ cells derived from RNA-seq (n=5, log2(Fold Change)=0.3, p=0.019, q=0.087). B) Stacked read plots analysis revealing high overlap between HLA-G and other selected HLA genes. C) RT-PCR validation of the transcriptional upregulation of HLA-G, IDO-1, CCL22 and CCL17 on G^+^ over G- T-cells (n=4-5 different donors from initial RNA-seq experiment, p<0.05).


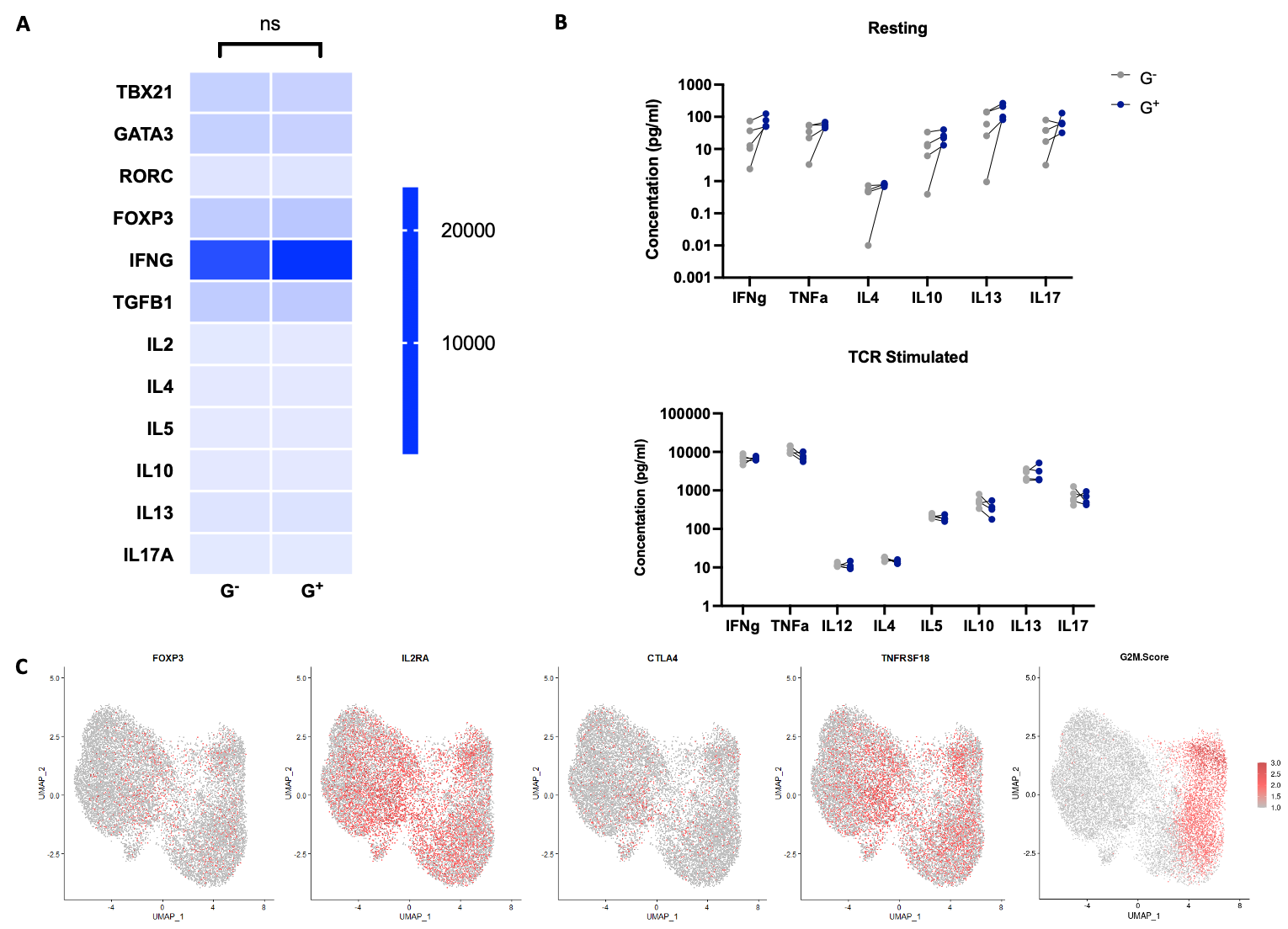


**Supplementary Figure 2. G^+^ cells do not comprise of established T-helper transcriptional signatures, albeit displaying molecular features akin to nTregs.** A) Heatmaps depicting transcriptional expression of key subtype-specific transcription factors and cytokines of G^+^ and G^-^ cells derived from the RNA-seq analysis. Colour grading (light to dark blue) indicates increasing levels of normalised reads derived from the DESEQ2 tool. B) Luminex assay for cytokine quantitative assessment in cell culture supernatants of G^+^ and G^-^ at rest (top) or following antiCD3/CD28 stimulation (bottom) presented in log scale (n=4-5). C) Expression of FOXP3, IL2RA (CD25), CTLA-4, TNFRSF18 (GITR) transcripts and G2/M cell-cycle gene signature score from the scRNA-seq of G^+^ cells overlaid on the UMAP (Uniform manifold approximation and projection) plot (shown in order from left to right).

**Supplementary Table 1. List of cell-type related marker genes used for cell-type annotation using the scCATCH algorithm**


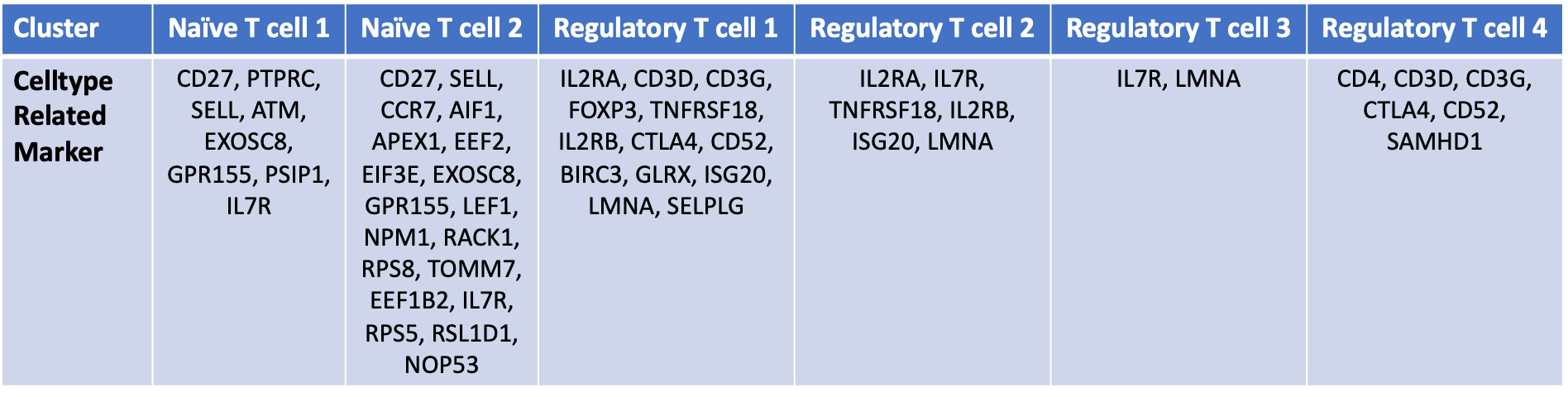


# References

1. InSyBio :: InSyBio ncRNASeq. Accessed February 24, 2023. https://www.insybio.com/ncRNASeq.html

2. Babraham Bioinformatics - FastQC A Quality Control tool for High Throughput Sequence Data. Accessed February 24, 2023. https://www.bioinformatics.babraham.ac.uk/projects/fastqc/

3. Bolger AM, Lohse M, Usadel B. Trimmomatic: a flexible trimmer for Illumina sequence data. *Bioinformatics*. 2014;30(15):2114-2120. doi:10.1093/bioinformatics/btu170

4. Kim D, Paggi JM, Park C, Bennett C, Salzberg SL. Graph-based genome alignment and genotyping with HISAT2 and HISAT-genotype. *Nat Biotechnol*. 2019;37(8):907-915. doi:10.1038/s41587-019-0201-4

5. Liao Y, Smyth GK, Shi W. featureCounts: an efficient general purpose program for assigning sequence reads to genomic features. *Bioinformatics*. 2014;30(7):923-930. doi:10.1093/bioinformatics/btt656

6. Love MI, Huber W, Anders S. Moderated estimation of fold change and dispersion for RNA-seq data with DESeq2. *Genome Biol*. 2014;15(12):550. doi:10.1186/s13059-014-0550-8

7. Jolliffe I. Principal Component Analysis. In: Lovric M, ed. *International Encyclopedia of Statistical Science*. Springer; 2011:1094-1096. doi:10.1007/978-3-642-04898-2_455

8. Cattell RB. The Scree Test For The Number Of Factors. *Multivariate Behav Res*. 1966;1(2):245-276. doi:10.1207/s15327906mbr0102_10

9. Thorvaldsdóttir H, Robinson JT, Mesirov JP. Integrative Genomics Viewer (IGV): high-performance genomics data visualization and exploration. *Briefings in Bioinformatics*. 2013;14(2):178-192. doi:10.1093/bib/bbs017

10. Huang DW, Sherman BT, Lempicki RA. Systematic and integrative analysis of large gene lists using DAVID bioinformatics resources. *Nat Protoc*. 2009;4(1):44-57. doi:10.1038/nprot.2008.211

11. Croft D, Mundo AF, Haw R, et al. The Reactome pathway knowledgebase. *Nucleic Acids Research*. 2014;42(D1):D472-D477. doi:10.1093/nar/gkt1102

12. Kanehisa M, Goto S. KEGG: kyoto encyclopedia of genes and genomes. *Nucleic Acids Res*. 2000;28(1):27-30. doi:10.1093/nar/28.1.27

13. Ashburner M, Ball CA, Blake JA, et al. Gene Ontology: tool for the unification of biology. *Nat Genet*. 2000;25(1):25-29. doi:10.1038/75556

14. Que X, Checconi F, Petrini F, Gunnels JA. Scalable Community Detection with the Louvain Algorithm. *2015 IEEE International Parallel and Distributed Processing Symposium*. Published online May 2015:28-37. doi:10.1109/IPDPS.2015.59

15. Becht E, McInnes L, Healy J, et al. Dimensionality reduction for visualizing single-cell data using UMAP. *Nat Biotechnol*. 2019;37(1):38-44. doi:10.1038/nbt.4314

16. Shao X, Liao J, Lu X, Xue R, Ai N, Fan X. scCATCH: Automatic Annotation on Cell Types of Clusters from Single-Cell RNA Sequencing Data. *iScience*. 2020;23(3):100882. doi:10.1016/j.isci.2020.100882

17. Bergen V, Lange M, Peidli S, Wolf FA, Theis FJ. Generalizing RNA velocity to transient cell states through dynamical modeling. *Nat Biotechnol*. 2020;38(12):1408-1414. doi:10.1038/s41587-020-0591-3

18. Dong M, Thennavan A, Urrutia E, et al. SCDC: bulk gene expression deconvolution by multiple single-cell RNA sequencing references. *Briefings in Bioinformatics*. 2021;22(1):416-427. doi:10.1093/bib/bbz166
